# Evaluation of TaqMan Array card (TAC) for the detection of 28 respiratory pathogens

**DOI:** 10.1186/s12879-020-05562-x

**Published:** 2020-11-10

**Authors:** Keke Liu, Hongbo Jing, Ying Chen, Xin Zheng, Hua Jiang, Decong Kong, Yvling Zheng, Shuiping Chen, Peng Liu, Yongqiang Jiang

**Affiliations:** 1 State Key Laboratory of Pathogen and Biosecurity, Institute of Microbiology and Epidemiology, Academy of Military Medical Sciences, Beijing, China; 2 Shandong Academy of Clinical Medicine, Shandong Provincial Hospital, Jinan, 250021 China; 3 Department of Laboratory Medicine, Shunyi District Center for Disease Control and Prevention, Beijing, China; 4 School of Food and Chemical Engineering, Beijing Technology and Business University, Beijing, 100048 China; 5 Department of Laboratory Medicine, the Fifth Medical Centre, Chinese PLA General Hospital, Beijing, China

**Keywords:** Respiratory pathogen, TaqMan Array card, Real-time PCR, Nucleic acid detection

## Abstract

**Background:**

Respiratory infections are a serious threat to human health. So, rapid detection of all respiratory pathogens can facilitate prompt treatment and prevent the deterioration of respiratory disease. Previously published primers and probes of the TaqMan array card (TAC) for respiratory pathogens are not sensitive to Chinese clinical specimens. This study aimed to develop and improve the TAC assay to detect 28 respiratory viral and bacterial pathogens in a Chinese population.

**Methods:**

To improve the sensitivity, we redesigned the primers and probes, and labeled the probes with minor groove binders. The amplification efficiency, sensitivity, and specificity of the primers and probes were determined using target-gene containing standard plasmids. The detection performance of the TAC was evaluated on 754 clinical specimens and the results were compared with those from conventional methods.

**Results:**

The performance of the TAC assay was evaluated using 754 clinical throat swab samples and the results were compared with those from gold-standard methods. The sensitivity and specificity were 95.4 and 96.6%, respectively. The lowest detection limit of the TAC was 10 to 100 copies/μL.

**Conclusions:**

TAC is an efficient, accurate, and high-throughput approach to detecting multiple respiratory pathogens simultaneously and is a promising tool for the identification of pathogen outbreaks.

## Background

Respiratory diseases, such as acute respiratory infections (ARIs) and community-acquired pneumonia (CAP), are a serious threat to human health [1–7]. Infection with unexplained respiratory pathogens further aggravates the mortality worldwide [8, 9]. Thus, rapid detection of all respiratory pathogens can facilitate prompt treatment and prevent the deterioration of respiratory disease [10]. Infections of viruses, bacteria, and mycoplasmas, chlamydia, and rickettsia often present similar respiratory symptoms and thus may lead to antibiotics abuse when the pathogens were not properly identified [7, 11–13]. Therefore, accurate identification of pathogens is essential for proper treatment of respiratory disease and avoidance of antibiotics abuse.

Bacterial culture, serology, ELISA, immunofluorescence staining, and conventional molecular diagnostics such as PCR and RT-PCR are common conventional approaches to detecting respiratory pathogens. These conventional detection methods have disadvantages; they have low sensitivity and are time-consuming, labor-intensive and susceptible to contamination [1, 3, 14]. The real-time PCR has been gradually applied to clinical diagnosis and show good results [15]. But it has limited application in multi-pathogen detection. Several molecular biological platforms for detecting multiple respiratory pathogens, such as FilmArray [16], RespiFinder [17] and other multiplex PCR detection systems have been developed [1, 3, 18, 19]. However, these multiplex platforms sometimes lead to false results, and modification of primers and probes for one pathogen requires re-optimization of all the primers and probes on the platforms [1]. The TaqMan array card (TAC) assay was developed to avoid amplicon contamination and to improve the detection efficiency for multiple pathogens [1, 20–22]. Next-generation sequencing (NGS) also provides technical solution for detecting the pathogen [23]. But it usually take 2–4 days to give the result. The TAC is a customizable 384-well microfluidic real-time PCR system. Primers and probes that are specific to the targets are pre-allocated on the card. The TAC allows for simultaneous detection of up to 48 targets in one specimen, thus offering an easy means for multiple-pathogen detection [20, 21]. Since the early model of TAC, which can detect 21 respiratory pathogen targets, was developed in early 2011 [1], several studies on the application of TAC to detect enteropathogens have been published and demonstrated its great effectivity to detect 19 enteropathogens [20, 24].

Although a number of articles have reported the use of the TAC for the detection of respiratory pathogens, comparison of its detection performance versus that of other diagnostic methods on large-scale clinical samples is still lacking [1, 18, 19, 21]. Moreover, the TAC released in 2011 had low sensitivity to some clinical specimens [1]. Further, the profile of prevalent respiratory pathogen strains in China may be different from the well-characterized profile that is used for the commercial TAC. Thus, the current study aimed to develop a broad-spectrum TAC to detect 28 prevalent respiratory pathogens in China and to compare the performance of the TAC assay with that of gold-standard methods including bacterial culture and real-time PCR. We anticipate that this novel method can be applied in China for pathogen detection, especially in surveillance and outbreak backgrounds.

## Methods

### Strains

Bacterial and viral strains that had been isolated from clinical specimens were used to validate the TAC. Briefly, the following 20 viruses and 8 bacteria were collected from other laboratories: influenza type A virus, influenza type B virus, enterovirus, parainfluenza virus (subtypes 1 to 3), respiratory syncytial virus (subtypes A and B), human metapneumovirus (subtypes A and B), adenovirus, rhinovirus, human bocavirus, human coronavirus (subtypes HCOV-229E, HCOV-NL63, HCoV-HKU, and HCOV-OC43), measles virus, mumps virus, rubella virus, *Mycoplasma pneumoniae*, *Chlamydia pneumoniae*, *Coxiella burnetii*, *Legionella pneumophila*, *Haemophilus influenza*, *Mycobacterium tuberculosis*, *Streptococcus pneumonia*, and *Bordetella pertussis*.

### Clinical specimens

Clinical specimens were obtained from the Affiliated Hospital of the Academy of Military Medical Sciences and the Center for Disease Control and Prevention of Shunyi District in Beijing, China. The protocol for collecting and handling of clinical specimens had been approved by the institutional review boards of both institutes. In total, 754 clinical specimens were collected between July 2013 and May 2015 from patients presenting respiratory syndromes including fever, cough, and runny nose. Nasopharyngeal/oropharyngeal (NP/OP) swabs were kept in 1 mL of universal transport medium and stored at − 80 °C. All of the 754 specimens were both tested by bacterial culture and individual real-time PCR. Three hundred seventy were positive, which most of them were identified by real-time PCR, and 384 were negative.

### Total nucleic acid extraction

Total nucleic acid was extracted from the clinical specimens (NP/OP swabs) and the bacterial and viral isolates using QIAamp cador Pathogen Mini Kit following the manufacturer’s instructions. Briefly, every sample was pretreatment with buffer ATL and pathogen Lysis L with glass beads according to the manufacturer’s instruction. Two hundred μL of the medium containing NP/OP swabs was mixed with lysis buffer containing 20 μL proteinase K and 100 μL buffer VXL with 1 μg carrier RNA and incubated at 25 °C for 15 min. Total nucleic acid from each sample was eluted in 80 μL buffer AVE. The extracted total nucleic acid was aliquoted and stored in − 80 °C for future use. A negative control (H_2_O) was included for each batch of extraction. If the negative control showed positive on the TAC assay, the entire batch of total nucleic acid was discarded.

### Plasmid construction

Standard plasmids were used to estimate the amplification efficiency and the limit of detection of the TAC assay. Plasmids containing 30 target genes, including 20 viral and 8 bacterial genes, and 2 internal positive controls were constructed and used as standard plasmids. The 28 target genes were amplified by PCR and the primers are list in Table 1. The amplified segments were inserted to the plasmid using the P-EASY-Blunt Zero Cloning Kit (Tran, Beijing, China). Recombinant plasmids were verified by sequencing. The RNA templates were first reverse-transcribed into cDNA, and then amplified and inserted into the plasmid of the cloning kit. The constructed plasmids were purified using the TIANprep Mini Plasmid Kit (Tran) according to the manufacturer’s instructions. The DNA concentration and quality of the purified recombinant plasmids was determined using a NanoDrop 2000 (Thermo Scientific, Waltham, MA). The number of copies per μL of each standard plasmid was calculated according to the equation as described previously [31].

**Table 1:** Primers and probes applied in this study

Pathogen	Target gene	Primer/probe sequence	Reference or Source
INF-A	*M*	F, AAGACCAATCCTGTCACCTCTGA	[2] (modified)
		R, AAGCGTCTACGCTGCAGTCC	
		P, ACGCTCACCGTGCC	
INF-B	*HA*	F, AAATACGGTGGATTAAACAAAAGCAA	[25] (modified)
		R, CCAGCAATAGCTCCGAAGAAA	
		P, TGGGCAATTTCCTATGGC	
PIV-1	*HN*	F, TGATTTAAACCCGGTAATTTCTCAT	[26]
		R, CCTTGTTCCTGCAGCTATTACAGA	
		P, ACGACAACAGGAAATC	
PIV-2	*HN*	F, AGGACTATGAAAACCATTTACCTAAGTGA	[26] (modified)
		R, AAGCAAGTCTCAGTTCAGCTAGATCA	
		P, TGTTCAGTCACTGCTATAC	
PIV-3	*HN*	F, AAAAGTTGATGAAAGATCAGATTATGCAT	[26] (modified)
		R, CCGGGACACCCAGTTGTG	
		P, AAAGGCAAAATAATATTTCTC	
HMPV-A	*F*	F, AGAGATGTAGGCACCACAACTGC	Beijing Genomics institution
		R, CTGATCCTAGAGCCGTGCAAA	
		P, TTCATCATTGCAGCAAGA	
HMPV-B	*F*	F, ACAATGGCAACTTTGCTTAAAGAA	Beijing Genomics institution
		R, GATTATAGGTGTGTCTGGTGCTGAA	
		P, ATATTCCACAAAATCAGAGGC	
HRV	*5′ UTR*	F, TTCCAGCCTGCGTGGC	[1] (modified)
		R, GAAACACGGACACCCAAAGTAGTC	
		P, CCCCTGAATGYGGC	
HEV	*5′ UTR*	F, GGTGYGAAGAGYCTATTGAGC	Beijing Genomics institution
		R, ACGGACACCCAAAGTAGTCG	
		R, TCCGGCCCCTGAAT	
HAdV	*Hexon*	F, CCCAGTGGTCTTACATGCACAT	[3] (modified)
		R, GCCACGGTGGGGTTTCTAA	
		P, CCGGGTCTGGTGCAG	
RSV-A	*N*	F, AGATCAACTTCTGTCATCCAGCAA	[25] (modified)
		R, TTCTGCACATCATAATTAGGAGTATCAAT	
		P, CGGAGCACAGGAGAT	
RSV-B	*N*	F, AAGATGCAAATCATAAATTCACAGGA	[27]
		R, TGATATCCAGCATCTTTAAGTATCTTTATAGTG	
		P, CTGGACATAGCATATAAC	
*M. pneumoniae*	*P1*	F, GCAGTTGCTGGCGCTAAGTT	Beijing Genomics institution
		R, AAGCGAGGTACGGTAGCGGTAT	
		P, TGGTAGGGAACTCGTTTTA	
*C. pneumoniae*	*MOMP*	F, CGTGGAGCCTTATGGGAATG	Beijing Genomics institution
		R, CGTCTGTTGGCAAGGGGA	
		P, CAGTCCAAACCTAAAGTT	
*S. pneumoniae*	*lytA*	F, ACGCAATCTAGCAGATGAAGCA	[1] (modified)
		R, TCGTGCGTTTTAATTCCAGCT	
		P, AACGCTTGATACAGGGAG	
*M. tuberculosis*	*orfB*	F, GGCTGTGGGTAGCAGACC	[28] (modified)
		R, CGGGTCCAGATGGCTTG	
		P, ACCTGGGCAGGGTT	
HBOV	*NP1*	F, AGAGGCTCGGGCTCATATCA	[29] (modified)
		R, CACTTGGTCTGAGGTCTTCGAA	
		P, CAATCARCCACCTATCGTCT	
Measles virus	*P*	F, GCAATTGGATCAACTGAAGGC	Beijing Genomics institution
		R, AGAGTCAGCATCTTGGATTCCCT	
		P, ACAGCGGTGAAGCG	
Rubella Virus	*E1*	F, ACGCCGCACGGACAACT	Beijing Genomics institution
		R, TGTTGGTTGCCGGTGTAATTC	
		P, AGGTCCCGCCCGAC	
Mumps virus	*P*	F, GCAATTGGATCAACTGAAGGC	Beijing Genomics institution
		R, AGAGTCAGCATCTTGGATTCCCT	
		P, ACAGCGGTGAAGCG	
*Coxiella burnetii*	*ICD*	F, AATTTGGAGCAAAGCCCTTAGA	Beijing Genomics institution
		R, GTAAAAAGGCGTCGGCAATAAC	
		P, ACCCTGGCATGTCT	
HCoV-229E	*N*	F, CAGTCAAATGGGCTGATGCA	[25] (modified)
		R, CAAAGGGCTATAAAGAGAATAAGGTATTCT	
		P, AACGTGGTCGTCAGGG	
HCoV-NL63	*N*	F, GCGTGTTCCTACCAGAGAGGAA	[25] (modified)
		R, GCTGTGGAAAACCTTTGGCA	
		P, TGCTTTGGTCCTCGTGAT	
HCoV-OC43	*N*	F, CGATGAGGCTATTCCGACTAGGT	[30] (modified)
		R, CCTTCCTGAGCCTTCAATATAGTAACC	
		P, TGGCACGGTACTCC	
HCoV-HKU	*N*	F, AGGGATCCTACTAYTCAAGAAGCTATCC	[3] (modified)
		R, ATGAACGATTATTGGGTCCACG	
		P, CGCCTGGTACGATTT	
Pan-Legionella	*5S–23S*	F, GTACTAATTGGCTGATTGTCTTGACC	[1] (modified)
		R, CCTGGCGATGACCTACTTTCG	
		P, ACTCTTTACCAAACCTG	
*H. influenzae*	*bexA*	F, GGACAAACATCACAAGCGGTTA	[1] (modified)
		R, TGCGGTAGTGTTAGAAAATGGTATTATG	
		P, TTGTAGTATTGATACGCTTTGT	
*B. pertussis* target I	*IS481a*	F, CAAGGCCGAACGCTTCAT	[1] (modified)
		R, GAGTTCTGGTAGGTGTGAGCGTAA	
		P, CCTTGCGTGAGTGGG	
*B. pertussis* target II	*PtxA*	F, GCCGCCAGCTCGTACTTC	[1] (modified)
		R, GGATACGGCCGGCATTG	
		P, CGTCGACACTTATGGCGA	
IPCO			ABI
RNP	*RPP30*	F, AGATTTGGACCTGCGAGCG	WHO (modified)
		R, GAGCGGCTGTCTCCACAAGT	
		P, CTGAAGGCTCTGCGC	

### Conventional detection methods

All clinical samples were tested by bacterial culture or individual real-time PCR. The sample was considered positive if one of the methods give the positive result. Moreover, in order to avoid contamination, all experiments involving conventional detection methods were performed at the Affiliated Hospital of the Academy of Military Medical Sciences and the Center for Disease Control and Prevention of Shunyi District in Beijing, China.

### Primer and probe design for TAC and TAC reaction conditions

**Fig. 1:**
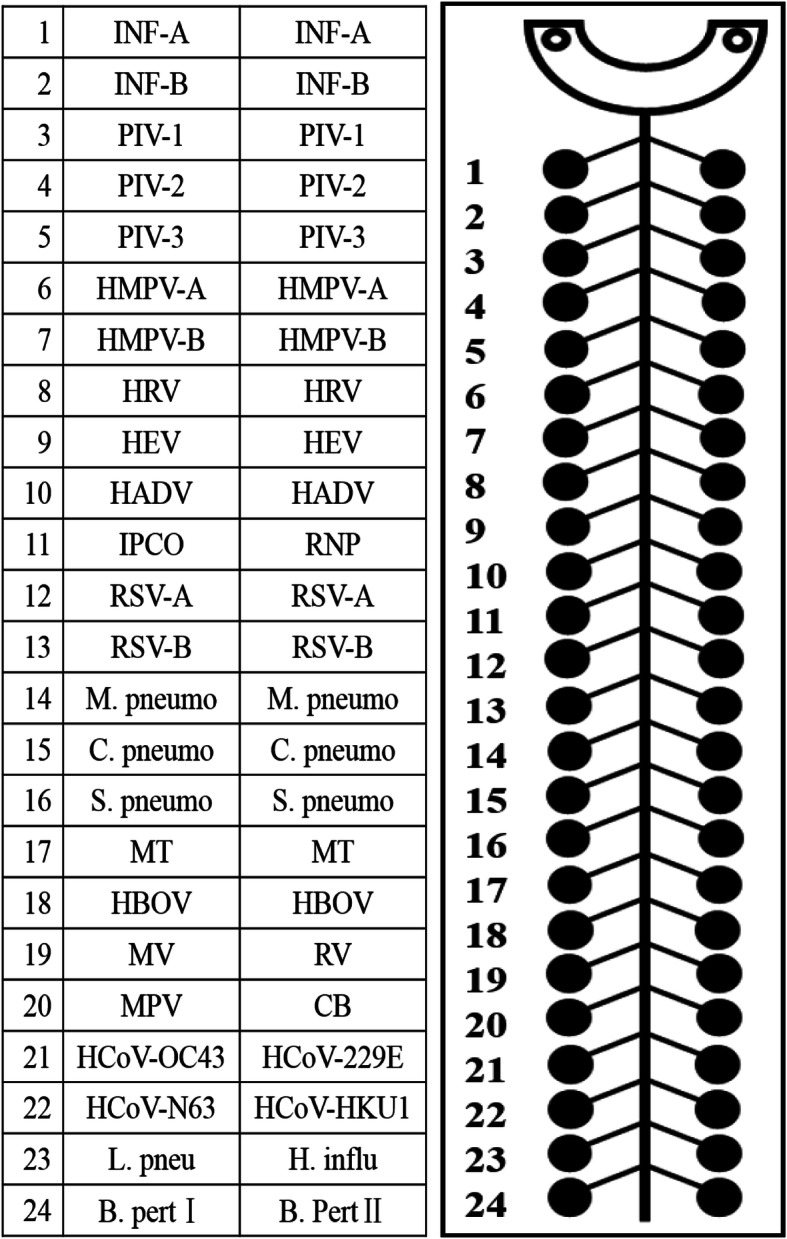
Respiratory TAC configuration. INF-A: influenza A; INF-B: influenza B; PIV-1/2/3: parainfluenza virus1/2/3; HMPV-A/B: human metapneumovirus A/B; HRV: human rhinovirus; HEV: human enterovirus; HAdV: human adenovirus; RSV-A/B: respiratory virus A/B; M. pneumo: *M. pneumoniae*; C. pneumo: *C. pneumoniae*; S. pneumo: *S. pneumoniae*; MT: *M. tuberculosis*; HBOV: Human bocavirus; MV: measles virus; RV: rubella virus; MPV: mumps virus; CB: *C. burnetii*; HCoV-OC43/229E/NL63/HKU: human coronavirus OC-43/229E/NL63/HKU L. pneumo: *Legionella pneumophila*; H. influ: *H. influenza*; B. pert I/II: *B. pertussis* target I/II. Each TAC contained one internal positive control (IPCO) and a clinical sample (RNP)

Primers and probes for 30 target genes (Fig. 1), designed using Primer Express 3.0 or adopted from published reports, were spotted onto the TAC. The primers and probes were designed to detect 28 respiratory pathogens, including a few subtypes, e.g., respiratory syncytial viruses (subtypes A and B), human metapneumovirus (subtypes A and B), parainfluenza virus (subtypes 1 to 3), and human coronavirus (subtypes HCOV-229E, HCOV-NL63, HCoV-HKU, and HCOV-OC43). Highly conserved regions were identified by multiple sequence alignment. To improve the specificity, we labeled probes with minor groove binders instead of black hole quenchers, which are commonly used in commercial TACs. The fluorophore of all probes are FAM. The target genes and the resources of primers and probes are displayed in Table 1.

The final primer and probe concentrations on the plate were 900 nM and 250 nM, respectively. The quantitative One Step qRT-PCR Kit (Tiangen, Beijing, China) was used and each 100-μL reaction mixture contained 50 μL 2× Quant One Step Probe qRT-PCR master Mix, 4 μL HotMaster Taq polymerase, 2 μL QuantRTase, 20 μL DNA/RNA, and 24 μL RNase-free water. The thermal cycling conditions were: 50 °C for 30 min, 92 °C for 3 min, and 40 cycles of denaturation at 92 °C for 10 s, annealing at 62 °C for 20 s, and elongation at 68 °C for 20 s. The PCR reactions were completed in a ViiA7 real-time PCR instrument (Life Technologies).

### Assessment of the amplification efficiency and detection limit of the primers and probes

Linearity and amplification efficiency were assessed as described previously [1, 21]. A 10-fold serial dilution of each target gene-containing standard plasmid (10^7^ to 10^3^ copies/μL) was prepared. Standard plasmids at two concentrations (high and low) were tested in triplicate to assess the intra- and interassay variability. The coefficient of variance (CV) was calculated based on cycle threshold (C_t_) values. The lowest detection limit (LOD) was defined as the lowest detectable concentration of standard plasmids. When ≥5 out of 7 replicates of a standard plasmid at 10^2^ copies/μL to 10^0^ copies/μL were detected, the test was considered positive.

### Evaluation of the TAC assay using clinical specimens

Out of the 754 clinical specimens, 370 tested positive and 384 negative in the gold-standard tests. All the negative samples were first processed on the ViiA7 to prevent contamination. RNase-free water was added to each card as a negative control template. A Ct cut-off value of 36 cycles was used to differentiate between positive and negative samples for all clinical specimens and the negative control on the TAC. The sensitivity and specificity of the TAC were evaluated by comparing the results from TAC assay with those from the gold-standard methods. The gold-standard methods were assumed to have 100% sensitivity and specificity. PCR products of 18 randomly chosen positive clinical specimens were sequenced to verify the accuracy of the TAC.

### Statistical analysis

Repeatability (three replicates within one card) and reproducibility (three replicates between cards) of the TAC are shown as CV values. Cohen’s κ was calculated to estimate the degree of consistency between TAC and the gold-standard methods by SPSS 17.0 software. Cohen’s κ was interpreted as follows: < 0, poor; 0–0.20, slight; 0.21–0.40, fair; 0.41–0.60, moderate; 0.61–0.80, substantial; and 0.81–1.00, almost perfect agreement [24]. *P* < 0.05 was regarded statistically significant.

### Ethics statement

The study was approved by the Fifth Medical Centre, Chinese PLA General Hospital and the Center for Disease Control and Prevention of Shunyi District in Beijing, China. Written informed consent was obtained from each patient or a guardian.

## Results

### The validation of TAC

The TAC is designed to detect 30 different target genes, including two replicates for influenza A, influenza B, parainfluenza virus1/2/3, human metapneumovirus A/B, human rhinovirus, human enterovirus, human adenovirus, respiratory virus A/B, *M. pneumoniae*, *C. pneumoniae*, *S. pneumoniae*, and *M. tuberculosis*, and one single for measles virus, rubella virus, mumps virus, *C. burnetii*, human coronavirus OC-43/229E/NL63/HKU, *L. pneumophila*, *H. influenza* and *B. pertussis* target I/II and two internal controls (Fig. 1). The primers and probes were designed to detect 28 respiratory pathogens, including 20 viruses (including different subtypes) and 8 bacteria. These pathogens are well recognized to cause respiratory symptoms in China [6].

The linearity and amplification efficiency of the primers and probes for 28 pathogens and two internal controls were analyzed using the standard plasmids. The linear coefficient (*r*^2^) for the 30 targets was between 0.990 and 1. The amplification efficiency was 90 to 105% and detection limit ranged from 5 to 50 copies/μL for the 28 pathogen target genes and one internal positive control target gene (Table 2). The intra-assay variation was 0.5 to 3% and the inter-assay variation assessed using 3 TACs was 1.5 to 10%.

**Table 2:** Validation of the TAC

Target	Linearity ***r***^**2**^ (amplification efficiency %)	Detection limit (copies/μL)
INF-A	0.998 (97.0)	10
INF-B	0.997 (99.0)	15
PIV-1	0.992 (99.2)	15
PIV-2	0.994 (97.3)	10
PIV-3	0.989 (95.6)	10
HMPV-A	0.998 (97.0)	10
HMPV-B	0.997 (93.7)	15
HRV	0.996 (95.0)	10
HEV	0.992 (103.6)	5
HAdV	0.995 (100.1)	10
RSV-A	0.998 (93.6)	10
RSV-B	0.999 (96.0)	10
*M. pneumoniae*	0.995 (97.3)	10
*C. pneumoniae*	0.998 (97.6)	50
*S. pneumoniae*	0.997 (94.7)	10
M.T	0.998 (93.5)	50
HBOV	0.995 (104.5)	10
MV	0.999 (99.0)	5
RV	1.000 (94.8)	5
MPV	0.998 (95.0)	15
CB	0.997 (96.2)	50
HCOV-229E	0.996 (95.1)	10
HCOV-NL63	0.997 (96.2)	10
HCOV-OC43	0.993 (97.7)	10
HCOV-KU1	0.995 (93.7)	10
PL	0.997 (94.5)	10
HI	1.000 (100.1)	10
*B. pertussis* I	0.998 (97.6)	15
*B. pertussis* II	0.997 (98.1)	15
RNP	0.999 (99.6)	10

In preliminary experiments, good amplification efficiency of primers and probes based on the analysis of standard plasmids did not always represent good amplification on clinical specimens. Thus, we optimized the primer and probes including their sequences and the concentrations spotted on the array using both the standard plasmids and clinical specimens.

### TAC performance for clinical specimens

The 754 clinical specimens contained 370 positive samples and 384 negative samples. All the specimens were tested with bacterial culture and individual real-time PCR. Most of the pathogens detected from the specimens were viruses. Compared with the results from the gold-standard methods, the diagnostic sensitivity of the TAC was 95.4% and the specificity was 96.6% (Table 3). The value of Cohen’s κ was 0.8 ~ 1.0, which indicated almost perfect agreement between the TAC assay and the conventional methods, except for human coronavirus (Table 4).

**Table 3:** Comparison of the results from TAC assay and those of the conventional methods

Pathogens	Established-method positive ***n*** = 370		Established-method negative ***n*** = 384	
	TAC+	TAC-	TAC+	TAC-
INF-A	51	4	2	35
INF-B	30	1	0	20
PIV-1	3	0	0	15
PIV-2	1	0	0	20
PIV-3	22	0	0	16
HMPV	26	0	0	40
HRV	33	4	2	23
HEV	21	1	2	20
HADV	18	2	1	34
RSV	62	2	0	19
MPN	18	0	3	36
SP	10	0	0	10
MT	15	0	0	15
HBOV	23	2	1	23
MV	13	0	0	15
RV	2	0	0	10
HCOV	5	1	2	20
Total	353	17	13	371
	Sensitivity = 95.4%		Specificity = 96.6%

**Table 4:** The kappa values between TAC and conventional methods

Pathogen	Kappa value	***P*** value
INF-A	0.866	<0.05
INF-B	0.959	<0.05
PIV-1	1.000	<0.05
PIV-2	1.000	<0.05
PIV-3	1.000	<0.05
HMPV	1.000	<0.05
HRV	0.801	<0.05
HEV	0.864	<0.05
HADV	0.881	<0.05
RSV	0.934	<0.05
MPN	0.883	<0.05
SP	1.000	<0.05
MT	1.000	<0.05
HBOV	0.878	<0.05
MV	1.000	<0.05
RV	1.000	<0.05
HCOV	0.700	<0.05

To assess the accuracy of the TAC assay, we randomly selected 16 specimens that were positive for pathogens according to the gold-standard tests and sequenced the corresponding amplified segments. The sequencing results showed that in 15 of the 16 specimens, the pathogen was accurately identified by the TAC assay (Table 5), with only one false negative result. Human coronavirus was not identified by the TAC in one specimen.

**Table 5:** Confirmation of TAC results by PCR and sequencing the amplified segment

PCR	TAC	Sequencing	Accuracy of TAC
HRV	HEV	HEV	100%
HRV	HEV	HEV	100%
HRV	HEV	HEV	100%
HRV	HEV	HEV	100%
HRV	Negative	Negative	100%
HADV	Negative	Negative	100%
HADV	Negative	Negative	100%
HADV	Negative	Negative	100%
HCOV	INF-A	H3N2	100%
HCOV	INF-A	H3N3	100%
HCOV	Negative	Negative	100%
HCOV	Negative	Negative	100%
HCOV	HCOV-OC43	HCOV-OC43	100%
HCOV	HCOV-OC43	HCOV-OC43	100%
HCOV	HCoV-HKU	HCoV-HKU	100%
HCOV	Negative	HCoV-HKU	0

## Discussion

ARIs and CAP are caused by viral or bacterial infection [1, 2, 6, 12, 14]. The TAC assay is a simple, sensitive, rapid, and high-throughput method to detect respiratory pathogens [1, 20, 21]. The use of TAC to detect respiratory pathogens has been reported previously [1, 20, 21, 32]. However, the detection performance of the TAC assay as compared with other methods remains unclear. Kodani et al. [1] and Geoffrey et al. [18] compared the performance of TAC assay with that of individual real-time PCR using identical primers and probes and found that TAC appeared less sensitive than the PCR with a sensitivity of 54–95% and a specificity of 98–99% for different clinical specimens. A recent report showed comparable performance of TAC assay and fast-track diagnostics for detection of 13 respiratory pathogens [19]. To develop a TAC that suits the respiratory pathogen profile in China, we designed primers and probes to detect 28 pathogens known to commonly cause infections in China. The performance of the TAC assay was evaluated using 754 clinical throat swab samples and the results were compared with those from gold-standard methods.

The 30 primers and probes used in this study to detect 28 respiratory pathogens commonly occurring in China were either newly designed or modified from published sequences. We analyzed the linearity, amplification efficiency, and the LOD of the microfluid system, which all revealed near-ideal performance. In addition, because in a preliminary study, the amplification efficiency of the primers and probes as determined using the standard plasmids was not always an indicator of their effectiveness for target detection in clinical samples, we optimized the primers and probes using both standard plasmids and clinical specimens. The following reasons may contribute to the inconsistency in amplification efficiency between standard plasmids and clinical specimens: 1) some factors in clinical samples may interfere in the interaction between primers and templates; 2) some pathogens may have unknown subtypes, which may not be amplified by the primers and probes that are designed for the known subtypes; 3) the copy number of pathogens in clinical specimens may be extremely low.

Compared with the gold-standard methods, the TAC assay showed consistent results. The sensitivity and specificity of the TAC assay was 95.4 and 96.6%, respectively. The sensitivity was higher than that in the previous studies of Kodani et al. and Geoffrey et al. There is a possible reason: In the previous studies, clinical specimens had been stored over long periods, which might have led to sample disintegration. In our study, we used short-term-stored clinical specimens obtained from out- and in-patients from 2013 to 2015.

We used individual real-time PCR as the gold-standard method to verify negative results from the TAC assay in clinical specimens. Some clinical specimens showed an extremely low level of pathogen infection according to the TAC assay, and the Ct of those specimens was very close to the Ct cutoff value of 36 [1, 21]. We considered these specimens as “suspicious positive specimens” when their Ct was between 36 and 38. Individual real-time PCR on these suspicious positive specimens using primers and probes identical to those of the TAC showed positive amplification of the target gene in all cases. These results suggest that the TAC is less sensitive than individual real-time PCR assays, as reported by Kodani et al. [1]. This may be explained by the small reaction volume (1 μl), containing only 0.2 μl nucleic acid, used in the TAC assay, while 2 μl of clinical samples was added to a 25 μl reaction volume for individual real-time PCR. However, in real clinical practice, such low-level infection is not significant for diagnosis.

Conventional diagnostic methods for respiratory diseases, such as bacterial culture, ELISA, PCR and agarose gel electrophoresis, are often associated with some disadvantages. For example, germiculture is usually time consuming, PCR and agarose gel electrophoresis may lead to inaccurate results. The individual real-time PCR is a sensitive and rapid method but has limit application in multiple-pathogens detection [1, 3]. A standard diagnostic method for all 28 pathogens detected in this study is currently unavailable. As respiratory diseases can spread quickly [6] and may lead to serious pandemics, rapid identification of respiratory pathogens is critical for disease control. The TAC assay can identify multiple respiratory pathogens in 3 h include the whole procedure of pretreatment, column isolation and amplification.

The current study was limited by the small number of positive clinical specimens for some of the tested infectious agents. Thus, validation of the primers and probes for those pathogens may appear challenging. We are currently collecting additional clinical specimens to further test the TAC. Nevertheless, our findings suggest that the TAC assay is a rapid, convenient, and high-throughput assay for simultaneous detection of multiple pathogens. Thus, the TAC may provide a promising method for efficient surveillance of outbreaks of respiratory pathogens in China.

## Data Availability

The datasets are available by request to the corresponding author.

## Abbreviations

TAC: TaqMan array card
CT: Cycle threshold
NP/OP: Nasopharyngeal/oropharyngeal
